# Poor outcome of octogenarians admitted to ICU due to periprosthetic joint infections: a retrospective cohort study

**DOI:** 10.1186/s12891-020-03331-0

**Published:** 2020-05-15

**Authors:** Emre Yilmaz, Alexandra Poell, Hinnerk Baecker, Sven Frieler, Christian Waydhas, Thomas A. Schildhauer, Uwe Hamsen

**Affiliations:** 1 Department of General and Trauma Surgery, BG University Hospital Bergmannsheil, Ruhr University Bochum, Bürkle-de-la-Camp-Platz 1, 44789 Bochum, Germany; 2 Medical Faculty of the University Duisburg-Essen, Essen, Germany

**Keywords:** Periprosthetic joint infection; infection, Intensive care unit, Elderly, Octogenarions

## Abstract

**Background:**

Even though surgical techniques and implants have evolved, periprosthetic joint infection (PJI) remains a serious complication leading to poor postoperative outcome and a high mortality. The literature is lacking in studies reporting the mortality of very elderly patients with periprosthetic joint infections, especially in cases when an intensive care unit (ICU) treatment was necessary. We therefore present the first study analyzing patients with an age 80 and higher suffering from a periprosthetic joint infection who had to be admitted to the ICU.

**Methods:**

All patients aged 80 and higher who suffered from a PJI (acute and chronic) after THR or TKR and who have been admitted to the ICU have been included in this retrospective, observational, single-center study.

**Results:**

A total of 57 patients met the inclusion criteria. The cohort consisted of 24 males and 33 females with a mean age of 84.49 (± 4.0) years. The mean SAPS II score was 27.05 (± 15.7), the mean CCI was 3.35 (± 2.28) and the most patient had an ASA score of 3 or higher. The PJI was located at the hip in 71.9% or at the knee in 24.6%. Two patients (3.5%) suffered from a PJI at both locations. Sixteen patients did not survive the ICU stay. Non-survivors showed significantly higher CCI (4.94 vs. 2.73; *p* = 0.02), higher SAPS II score (34.06 vs. 24.32; *p* = 0.03), significant more patients who underwent an invasive ventilation (132.7 vs. 28.1; *p* = 0.006) and significantly more patients who needed RRT (4.9% vs. 50%; *p* < 0.001). In multivariate analysis, RRT (odds ratio (OR) 15.4, CI 1.69–140.85; *p* = 0.015), invasive ventilation (OR 9.6, CI 1.28–71.9; *p* = 0.028) and CCI (OR 1.5, CI 1.004–2.12; *p* = 0.048) were independent risk factors for mortality.

**Conclusion:**

Very elderly patients with PJI who needs to be admitted to the ICU are at risk to suffer from a poor outcome. Several risk factors including a chronic infection, high SAPS II Score, high CCI, invasive ventilation and RRT might be associated with a poor outcome.

## Background

The term “silver tsunami” describes the progressively ageing population in developed countries and the huge socioeconomic shift that is expected to effect various clinical fields including the „rise and burden of hip and knee osteoarthritis “[1]*.* According to Kiadaliri et al. the number of prevalent osteoarthritis cases increased by 43% between 1990 and 2015. The number of knee osteoarthritis alone has doubled in prevalences since the mid-twentieth century [2]. Thus, the estimated prevalence of Total Hip (THR) and Total Knee Replacements (TKR) in the United States was 2,552,815 and 4,700,621 respectively in 2010. Out of these 640,740 (THR) and 1,087,400 (TKR) were at the age of 80 and higher [3].

Knee and hip arthroplasty are a succesful treatment for osteoarthritis in terms of pain relief, function recovery and enhancing life quality [4, 5]. Even though surgical techniques and implants have evolved, periprosthetic joint infection (PJI) remains a serious complication leading to poor postoperative outcome and a high mortality [6]. The incidence of PJI after total joint arthroplasty differs according to localization and type between 1 and 3% [7]. Up to now, there is no gold standard treatment for patients with PJI. In addition to the Musculoskeletal Infection Society (MSIS) criteria introduced by the American Academy of Orthopedic Surgeons (AAOS) in 2011 [8, 9], the concept of Trampuz and Zimmerli is well known in Europe [10, 11]. Furthermore, treatment of PJI often includes a prolonged hospital stay, multiple surgeries, prolonged antimicrobial treatment, protheses and medical supplies which can lead to a 24 times higher treatment cost [12, 13]. Several different risk factors have been described for PJI after Total Joint Arthroplasty including obesity, urinary tract infection, diabetes and rheumatoid arthritis [6]. However, the literature is conflicted when it comes to determine age as an independent risk factor. In developed countries the proportion of elderly patients admitted to the Intensive Care Unit (ICU) increased dramatically [14, 15]. The literature is lacking in studies reporting the mortality of very elderly patients with periprosthetic joint infections, especially in cases when an ICU treatment was necessary. We therefore present the first study analyzing patients with an age 80 and higher suffering from a periprosthetic joint infection who had to be admitted to the ICU.

## Methods

The study has been approved by the local Ethical Committee (No. of approval 18–6260-BR). From January 2012 and December 2016, all patients aged 80 and higher who suffered from a PJI (acute and chronic according to the definition as described by Li et al. [16]) after THR or TKR and who have been admitted to the ICU have been included in this retrospective, observational, single-center study. In defining periprosthetic joint infection all patients fullfilled criteria according to the European Bone and Joint Infection Society (EBJIS) and Musculoskeletal Infection Society (Table 1) [8, 9, 17]. The ICU consisted of 13-bed surgical intensive care unit in a Level 1 university and referral hospital for PJI in Germany. The ICU is accompanied by a stand-alone Intermediate Care Unit (IMC). The IMC ressourses and therapeutic options include an intensivist-led 24 h presence of a resident experienced in intensive care, monitoring corresponding to ICU-standard, non-invasive ventilation and continuous vasopressor-administration. Therefore, most surgical patients suffering PJI at risk or not stable enough for normal ward are admitted to the IMC. Severity of illness were assessed using the Simplified Acute Physiology Score II (SAPS II) [18], the American Society of Anaesthesiologists Score (ASA) [19] and the Charlson Comorbidity Index (CCI) [20].

**Table 1:** Definition of Periprosthetic Joint Infections according to the EBJIS criteria and Musculoskeletal Infection Society

*EBJIS criteria*	
I	Clinical: sinus tract (fistula) or purulence around prosthesis
II	Cell count in joint aspiration: > 2000/μl leukocytes or > 70% polymorphonuclear granulocytes (PMN)
III	Histology: inflammation in periprosthetic tissue (type 2 or 3 after Krenn Morawietz)
IV	Microbial growth in synovial fluid or > = 2 tissue samples (in cases of high virulent microbes like *Staphylococcus aureus* one sample is considered sufficient) or sonication fluid ≥50 CFU/ml
A PJI is diagnosed if at least one of the following criteria is fullfilled	
**Musculoskeletal Infection Society criteria**	
**Definition of Periprosthetic Join Infection According to the International Consensus Group. This Is An Adaptation of the Musculoskeletal Infection Society Definition of PJI.**	
PJI Is Present When One of the Major Criteria Exists or Three Out of Five Minor Criteria Exist	
**Major Criteria**	
Two positive periprosthetic cultures with phenotypically identical organisms, OR	
A sinus tract communicating with the joint, OR	
**Minor Criteria**	
1) Elevated serum C-reactive protein (CRP) AND erythrocyte sedimentation rate (ESR)	
2) Elevated synovial fluid white blood cell (WBC) count OR ++change on leukocyte esterase test strip	
3) Elevated synovial fluid polymorphonuclear neutrophil percentage (PMN%)	
4) Positive histological analysis of periprosthetic tissue	
5) A single positive culture	

### Statistical analysis

Data were analyzed using SPSS version 21.0 (SSPS Inc., Chicago, IL) and Excel version 16.16.7 (Microsoft Corporation, Redmond, WA, USA). Univariate analysis was performed to compare demographics, surgical characteristics, and intensive care treatment. For categorical variables, frequency counts were computed and presented along with their percentages. For continuous variables, means were computed and presented along with their range. Mann Whitney U-test or Student’s T-Test were used, as appropriate. Statistical significance was set at *p* < 0.05. Multivariate analysis (binary logistic) was performed using the four most significant parameters in univariate analysis to determine independent risk factors for mortality.

## Results

A total of 57 patients met the inclusion criteria. The cohort consisted of 24 males and 33 females with a mean age of 84.49 (± 4.0) years. The mean SAPS II score was 27.05 (± 15.7), the mean CCI was 3.35 (± 2.28) and the most patient had an ASA score of 3 or higher. The PJI was located at the hip in 71.9% or at the knee in 24.6%. Two patients (3.5%) suffered from a PJI at both locations. Most patients suffered from a chronic infection (86%) and underwent a planned surgical intervention (50.9%). The results are summarized in Tables 2, 3, 4.

**Table 2:** Patient demographics are summarized in Table 2

	*n* = 57 (Mean ± SD) n (%)
Baseline Factors	
Age (years)	84.49 ± 4.0
Sex (male)	24 (42.1%)
BMI*	26.70 ± 5.25
SAPS II*	27.05 ± 15.7
CCI*	3.35 ± 2.28
ASA Score*	3.09 ± 0.58
ASA Score ≥ 3*	40 (87.8%)

*SAPS II Simplified Acute Physiology Score II; *CCI* Charlson Comorbidity Index; *ASA* American Society of Anaesthesiologists Score; *BMI* Body mass index

**Table 3:** Prosthetic Joint Infection locations are summarized in Table 3

	*n* = 57 (Mean ± SD) n (%)
Hip	41 (71.9%)
Knee	14 (24.6%)
Hip and Knee	2 (3.5%)
Acute Infection (< 4 weeks)	8 (14%)
Chronic Infection (> 4 weeks)	49 (86%)
Number of surgical interventions since prothesis implantation	2.19 ± 3.2 (0.10)

**Table 4:** Reasons for ICU admission are summarized in Table 4

	n = 57 (Mean ± SD) n (%)
Planned surgical intervention	20 (50.9%)
Medical Reason	12 (21.1%)
Unplanned surgical intervention	16 (28.1%)
Transfer from other ICU	7 (12.3%)

Sixteen patients did not survive the ICU stay. In univariate analysis, non-survivors showed significantly higher CCI (4.94 vs. 2.73; *p* = 0.02), higher SAPS II score (34.06 vs. 24.32; *p* = 0.03), significant more patients who underwent an invasive ventilation (132.7 vs. 28.1; *p* = 0.006) and significantly more patients who needed renal replacement therapy (RRT) (4.9% vs. 50%; *p* < 0.001). Results are summarized in Table 5. In multivariate analysis, RRT (odds ratio (OR) 15.4, CI 1.69–140.85; *p* = 0.015), invasive ventilation (OR 9.6, CI 1.28–71.9; *p* = 0.028) and CCI (OR 1.5, CI 1.004–2.12; *p* = 0.048) were independent risk factors for mortality (Table 6).

**Table 5:** Factors associated with poor mortality are summarized in Table 5

	survivor	non-survivor	***p***-value
cases	41 (71.9%)	16 (28.1%)	
Male gender	17 (41%)	7 (43%)	0.87
Age, mean ± SD	83.8 ± 3.3	86.2 ± 5.2	0.1
Days on ICU, mean ± SD	10.1 ± 11.1	16.4 ± 16.5	0.1
CCI*, mean ± SD	2.73 ± 2.04	4.94 ± 2.14	**0.02**
ASA score*, mean ± SD	3.12 ± 0.51	3.00 ± 0.73	0.54
SAPS II Score*, mean ± SD	24.32 ± 15.3	34.06 ± 14.8	**0.03**
BMI*, mean ± SD	27.64 ± 5.65	24.06 ± 2.55	**0.03**
Invasive ventilation, no(%)	8 (20%)	9 (56%)	**0.006**
Hours of ventilation, median ± SD	28.1 ± 41.9	132.7 ± 143.3	0.06
RRT*, no (%)	2 (4.9%)	8 (50%)	**< 0.001**
Number of surgical intervention during hospital stay	2.3 ± 1.9	1.9 ± 1.5	0.3
Number of surgical intervention since prosthesis implantation, mean ± SD	2.8 ± 3.5	0.7 ± 1.1	0.3
**Localisation of PJI**			
Hip, no (%)	28 (68%)	13 (81%)	0.5
Knee, no (%)	11 (27%)	3 (19%)	0.5
Knee and Hip	2 (5%)	0	0.5
Transferred from other ICU	2 (5%)	5 (31%)	**0.006**
**Reason for ICU admission**			
Unplanned surgical	14	2	0.26
Unplanned medical	8	4	0.26
Scheduled surgical	19	10	0.26
Acute Infection	7	1	0.29

* *CCI* Charlson Comorbidity Index; *ASA* American Society of Anaesthesiologists Score; *BMI* Body mass index; *RRT* Renal replacement therapy; *SAPS II* Simplified Acute Physiology Score II; *PJI* Periprosthetic joint infection

**Table 6:** Multivariate logistic regression for mortality is shown in Table 6*

	OR*	95%-CI*	***p***-value
CCI*	1.5	1.004–2.12	**0.048**
Invasive ventilation	9.6	1.28–71.9	**0.028**
RRT*	15.4	1.69–140.85	**0.015**
Transferred from other ICU	2.5	0.39–15.47	**0.339**

* *OR* Odds ratio; *CI* Confidence interval; *CCI* Charlson Comorbidity Index; *RRT* Renal replacement therapy

## Discussion

This study presents the first study ever to analyze the outcome of octagenarions in the setting of PJI and ICU treatment. PJI is a devastating complication resulting in severe pain, functional impairment and high mortality [21]. Furthermore, the estimated costs for infection revision is expected to be as high as $ 1.62 billion in the United States alone [22]. A validated risk score to assess and predict PJI does not exist. However, several risk factors have been discussed in the setting of PJI. Zuh et al. reported in their systematic review that body mass index, diabetes mellitus, corticosteroid therapy; hypoalbuminaemia, rheumatoid arthritis, blood transfusion, presence of a wound drain, wound dehiscence, superficial surgical site infection, coagulopathy, malignancy, immunodepression, National Nosocomial Infections Surveillance (NNIS) score ≥ 2, prolonged operative time and previous surgery are potential risk factors for PJI [23].

Even though most of these factors were not analyzed in detail in our study the vast majority of our patients had an ASA score of 3 and higher. Maaloum et al. reported a mortality rate of 20% in their retrospective case series analyzing 41 patients (mean age: 71.8 ± 9.4 years) suffering from a PJI admitted to the ICU. They could show as well that a high SAPS II score and a high ASA score is associated with a high mortality rate [24]. We also observed a significantly higher CCI in non-survivors compared to patients who have survived (4.94 ± 2.14 vs. 2.73 ± 2.04; *p* = 0.02). The proportion of patients requiring a RRT (50% vs. 4.9%; *p* < 0001) or invasive ventilation (56% vs. 20%; *p* = 0.006) was significantly higher in the non-survivor group in our study. These findings have been reported by several studies [25] and the same trends were observed by Maaloum et al. with more patients requiring RRT (50% vs, 15%; *p* = 0.05) or mechanical ventilation (88% vs. 76%; *p* = 0.66) in the non-survivor group [24].

The literature is conflicted with respect to determine age as an independent risk factor on survival in elderly patients. Martin-Loeches et al. reported in their prospective multicenter study that septic patients aged 80 and over have a higher hospital mortality compared to patients younger than 80 [14]. However, Flaatten et al. could show that the Clinical Frailty Scale is inversely associated with the 30-day survival. While 76% of the patients classified as “fit” were estimated to survive at 30 days following ICU admission only 59% of the patients who were classified as “frail” were estimated to survive the 30-day follow-up [26]. Our results also suggest that age per se has a smaller impact on survival than other factors such as the CCI, SAPS II and RRT [26, 27].

We observed a significantly higher rate of patients transferred from another ICU (31% vs. 5%; *p* = 0.006) in the non-survivor group. This might be explained by a delayed therapy, especially in cases when the septic prothesis has not been removed in the transferring hospital or the adequate antibiotic treatment has not been started. We did not analyze the surgical treatment delay in patients who have been transferred to our ICU. Nevertheless, previous studies have shown that an immediate treatment within the first hours is associated with a reduction in hospital mortality in very old patients [14, 28].

Treatment of very elderly patients admitted to the ICU is complex and represents an ongoing challenge for surgeons and intensive care specialists. Even though systematic ICU admissions of elderly patients failed to reduce the mortality [29] an appropriate and systematic approach with precise predictions models are needed for this patient group [30, 31].

### Limitations

This study has several limitations: It is an observational, non-comperative, single-center cohort study in a retrospective setting, and therefore we may have missed data points and there is potential for bias or residual confounding from factors we did not measure. The available literature is lacking in comparible studies. Furthermore, there is a huge variety in the definition of acute and periprosthetic joint infections in the literature ranging from 4 up to 12 weeks. Therefore, conclusion based on our results should be drawn carefully.

However, this study is the first ever to report and analyze risk factors on survival in very elderly patients with PJI admitted to the ICU. More studies are warranted to better understand risk factors on mortality rates and offer these special patients the best possible treatment.

## Conclusion

Very elderly patients with PJI who needs to be admitted to the ICU are at risk to suffer from a poor outcome. Several risk factors including a chronic infection, high SAPS II Score, high CCI, invasive ventilation and RRT might be associated with a poor outcome. Health care providers should inform these patients accordingly. The literature is lacking in studies analyzing this particular group of patients and further research is needed. Prospective multi-center cohort trials and comparative clinical trials represent a key area of opportunity for future studies.

## Data Availability

The data used and analyzed during the current study are available in anonymized form from the corresponding author on reasonable request.

## Abbreviations

AAOS: American academy of orthopedic surgeons
ASA: American society of anaesthesiologists score
CCI: Charlson comorbidity index
EBJIS: European bone and joint infection society
ICU: Intensive care unit
IMC: Intermediate care unit
MSIS: Musculoskeletal infection society
NNIS: National nosocomial infections surveillance
OR: Odds ratio
PJI: Periprosthetic joint infection
RRT: Renal replacement therapy
SAPS II: Simplified acute physiology score II
THR: Total hip replacement
TKR: Total knee replacement
